# AuNP-Containing Hydrogels Based on Arabinogalactan and κ-Carrageenan with Antiradical Activity

**DOI:** 10.3390/gels12010009

**Published:** 2025-12-22

**Authors:** Marina Zvereva

**Affiliations:** A.E. Favorsky Irkutsk Institute of Chemistry, 664033 Irkutsk, Russia; mlesnichaya@mail.ru

**Keywords:** gold nanoparticles, polysaccharides, hydrogels, free radicals, chemiluminescence antiradical activity

## Abstract

The synthesis and the antiradical activity of composite hydrogels based on arabinogalactan-stabilized gold nanoparticles (AuNPs) and κ-carrageenan (κCG) were examined. It was found that the reducing and stabilizing properties of arabinogalactan (Ar-Gal) could be used to obtain hybrid composites. The AuNP content of the composites ranged from 0.3 to 7.8%, with an average particle size of 5.3–14.0 nm. The antiradical properties of these nanocomposites were demonstrated for the first time using the activated chemiluminescence method. The combination of the synthesized Ar-Gal-AuNPs composite and the natural gel-forming polysaccharide κCG at different ratios enabled a variety of composite hydrogels with different rheological and structural-mechanical properties to be produced. The effect of the Ar-Gal-AuNPs/κCG ratio and the presence of Ca^2+^ ions on the moisture-retaining ability, swelling properties and elastic modulus of the obtained hydrogels was investigated.

## 1. Introduction

Over the last decade, the scientific community has demonstrated a heightened level of interest in the synthesis of gold nanoparticles (“AuNPs”). This interest can be attributed to the prospects of their application in biomedical fields, particularly as optical sensors of biospecific interactions [1], agents for targeted drug delivery [2], in photothermolysis of cancer cells [3], photodynamic therapy [4], etc. [5,6,7]. Furthermore, the utilization of AuNPs for therapeutic purposes, particularly in the treatment of autoimmune diseases, has become widespread [8,9,10]. In these cases, positive results are associated with increased antiangiogenic activity due to the AuNPs binding with vascular endothelial growth factor, resulting in decreased tissue infiltration by macrophages and inflammation control [11]. AuNPs are also considered an effective means of treating inflammation. They achieve their therapeutic effect by reducing the level of pro-inflammatory cytokines (TNF-α, IL-1b, IL-6), stimulating the production of anti-inflammatory cytokines (IL-4), and inhibiting key inflammatory enzymes such as cyclooxygenase-2 [12,13]. Given the high medicinal potential of AuNPs in medicine, it is important to consider their ability to inhibit the excessive generation of free radicals, which are constantly formed in a normally functioning living organism [14]. At the same time, the neutralization of reactive oxygen species (ROS) and the suppression of oxidative stress are crucial for treating degenerative diseases and promoting wound healing. Due to their small size, AuNPs have a high surface-to-volume ratio and excess surface energy, resulting in high physicochemical activity of nano-objects [15]. The ability of gold nanoparticles to neutralize radical particles has been demonstrated in several studies [16,17,18,19]. The majority of these experiments were performed using spectrophotometric approaches. In particular, the pronounced antioxidant activity of AuNPs has been shown through the decolorization of free radicals, such as DPPH· and ABTS^+·^, or in vivo by stabilizing the levels of endogenous antioxidant enzymes (SOD, GSH, catalase, and GPx) and lipid peroxidation products in blood and biological tissues in response to the administration of AuNPs [20,21,22,23,24,25,26]. However, determining the radical-binding ability of potential antioxidants using systems based solely on DPPH· and ABTS^+·^ (as is mainly presented in the literature) is not specific to the processes occurring in vivo. This is due to factors ranging from the insolubility of DPPH· in water to the difference in the kinetics of interaction between these radicals and the actual pro-oxidant substances present in biological tissues. When investigating the antiradical activity of substances with potential biomedical applications (in this case, AuNPs), the conditions should resemble biomimetic ones as closely as possible (i.e., aqueous or buffered media with a natural pH, temperature and radicals that can be generated in biological media). Of the most widespread methods of studying the radical-binding capacity, chemiluminescent analysis is a notable example [27,28]. This method has several advantages over the frequently used photometric, chemical and electrochemical techniques, including its high sensitivity and specificity in modelling experimental conditions similar to natural biological ones. It also allows for a detailed study of the rate of reactions involving free radicals and the acquisition of information about the activity and antioxidant capacity of a potential antioxidant.

To date, a significant practical problem with the use of nanomaterials in biomedical applications is maintaining their nanodispersity and stability [29]. Special stabilizing agents of synthetic (polyethylene glycol, polyvinylpyrrolidone, polyethylenimine, etc.) or natural (polysaccharides, proteins, complex plant extracts) origin are utilized to preserve the nanoscale level of dispersion of gold particles [30]. In this work, the natural heteropolysaccharide Ar-Gal was employed as a stabilizing agent. Previous studies have already demonstrated the effectiveness of Ar-Gal as a stabilizing agent for a variety of nanoparticles [31]. Furthermore, when obtaining noble metal nanocomposites, Ar-Gal has proven to be an effective metal ion reducer [32,33]. This feature enables water-soluble stable nanoparticles of noble metals to be obtained from their inorganic salt precursors in one stage using only Ar-Gal, which acts both as a reducing agent and a stabilizer of the forming nanoparticles. Using Ar-Gal as a stabilizing shell for the nanoparticles allows the production of non-toxic nanomaterials with pronounced biological properties, which are determined by both the polysaccharide (e.g., biocompatibility, membrane-tropism, and hypolipidemic activity) and the nanoparticles themselves (e.g., antimicrobial, antiviral, antitumor, immunosuppressive, and anti-angiogenic properties). These nanomaterials can be produced with a high degree of reproducibility [34,35]. The biological activity and antioxidant properties of AuNPs, as well as water solubility and biological properties of polysaccharide Ar-Gal enables the development of advanced nanomaterials for controlling infectious, oncological, and autoimmune diseases. These nanomaterials can also compensate for the effects of excess free radical generation during the immune response to pathological progression. At the same time, the ease of use and prolonged action of Ar-Gal-stabilized AuNPs can be ensured by incorporating them in matrix of a gel-forming agent [36]. In particular, such an agent may be κCG, effectiveness of which has already been proven in the development of silver-containing hydrogels [37]. κCG’s partial depolymerization makes it possible to regulate its molecular weight and gelation parameters, while the introduction of metal cations as crosslinking agents affects the rate of gelation and the rheological and mechanical parameters of the resulting hydrogels. This makes it possible to obtain a number of biodegradable, non-toxic hydrogels based on κCG. The combination of κCG with Ar-Gal, which includes AuNPs in its composition, allows the rheological and structural-mechanical parameters of the resulting hydrogels to be varied. This ensures the directivity and convenience of their local application, as well as optimizing the duration of their action. This includes the ability to absorb wound exudate, ensuring the wound surface remains hygienic, and the slow release of active AuNPs, which relieve inflammation and balance the oxidative stress processes. At the same time, the resulting hydrogel compositions based on these two polysaccharides can have a high water-holding capacity and be non-toxic and biodegradable.

The aim of this work is to synthesize and structurally characterize the Ar-Gal-stabilized AuNPs in detail, as well as to evaluate their radical-binding capacity using a chemiluminescent approach. The work also focuses on the investigation of rheological and structural-mechanical properties of composite hydrogels based on the obtained Ar-Gal-stabilized AuNPs and κCG.

## 2. Results and Discussion

### 2.1. Synthesis of Ar-Gal-Stabilized AuNPs

The stability of the obtained AuNPs is determined by their incorporation into the macromolecules of Ar-Gal, a natural heteropolysaccharide extracted from Siberian larch. The Ar-Gal macromolecules have a branched structure, with the main chain consisting of β-1,3-linked D-galactopyranose residues. Most galactose residues have side branches at C-6. Meanwhile, 3,6-di-O- and 6-O-substituted β-L-arabinofuranose residues are the most common side branches [38]. A number of water-soluble hybrid organic-inorganic nanocomposites containing particles of noble metals, elemental chalcogens, metal oxides, chalcogenides, and other inorganic compounds as nanophase have been previously synthesized and characterized using Ar-Gal [34,35].

**Figure 1:**
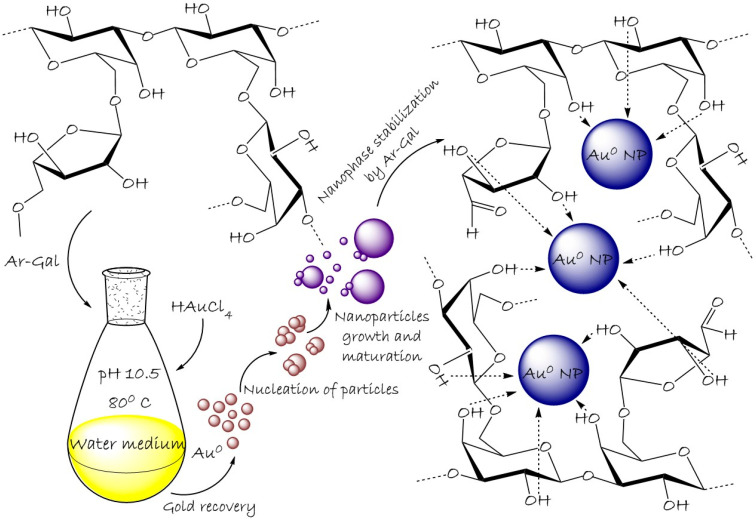
Suggested scheme for the formation of AuNPs in Ar-Gal matrix.

The nanocomposites containing Ar-Gal-stabilized AuNPs were synthesized using the previously described method for the formation of nanoparticles in galactose-containing polysaccharide matrices [31]. In particular, the interaction between Au^3+^ ions and Ar-Gal macromolecules in an aqueous medium afforded a number of water-soluble nanocomposites (Figure 1). These nanocomposites incorporate Ar-Gal and AuNPs in amounts ranging from 0.3% to 7.8%. At the same time, Ar-Gal macromolecules function as both a reductant, reducing the gold ions to the zero-valent state, and a stabilizer of the formed nanoparticles. This avoids the need for environmentally unfriendly reducing agents and solvents in the nanoparticle synthesis, and satisfies the basic requirements of ‘green’ chemistry. As with gold-containing nanocomposites, the quantitative content of Au in the nanocomposites could be varied by changing the Au^3+^/Ar-Gal ratio. The reaction was initiated by raising the pH of the reaction medium to 10.1–10.6 and the temperature to 80 °C. The synthesis time varied from 20 to 50 min depending on the amount of gold introduced.

It was found that the formation of polysaccharide-stabilized AuNPs is most effective at pH values greater than 10. This dependence of gold reduction on pH can probably be attributed to the formation of additional reducing monosaccharide fragments and intermediate compounds in an alkaline medium, resulting from the “peeling” of Ar-Gal macromolecules in an alkaline environment. This process can also involve the direct participation of hydroxide anions in binding protons generated during the redox reaction. Additionally, the reduction of gold ions in the Ar-Gal matrix may be facilitated by the direct oxidation of terminal aldehyde groups, which are present in small amounts (up to 0.4%) in the Ar-Gal composition, or of primary hydroxyl groups. Presumably, nucleation of a new solid phase of nanoparticles occurs due to the condensation of reduced atomic gold when the critical concentration is reached in the reaction medium. Further, the nanoparticles are formed through the continuous growth of the generated nuclei owing to the sorption of reduced gold atoms on their surface. This process is accompanied by the re-dissolution of unstable nuclei and the incorporation of released gold atoms into the lattice of the growing particles. Completion of the process is presumably dependent on the adsorption of Ar-Gal polysaccharide macromolecules onto the surface of the growing nanoparticle (Figure 1). The formation of AuNPs in the Ar-Gal matrix likely proceeds via a homogeneous phase formation mechanism, akin to that described earlier for the formation of selenium nanoparticles in the Ar-Gal matrix [35]. Thus, when the critical concentration of reduced Au0 atoms in the reaction medium exceeds the solubility limit, the atoms spontaneously combine to form new phase nuclei, in the form of Au^0^ clusters with a radius of approximately 1.1 nm, as reported in various literature sources. Particles smaller than this size undergo overgrowth, and the resulting released atoms are sorbed onto the surface of more stable growing particles. As the size of the nanoparticles increases, their solubility decreases. This can ultimately lead to a loss of aggregative and kinetic stability and the separation of the resulting gold from the reaction medium in the form of a massive precipitate. In this case, the active growth of nanoparticles is inhibited and their stability is ensured by the use of Ar-Gal. Its spatially branched, functionally saturated macromolecules passivate the surface of the formed AuNPs, ensuring their stability. At the same time, the adsorption of Ar-Gal onto the surface of growing gold nanoparticles limits the diffusion of Au0 atoms, consequently stopping their growth. Additionally, a sufficiently large shell of hydrated Ar-Gal macromolecules on the surface of the nanoparticles creates barriers to their fusion and the formation of aggregates. This creates conditions for the formation of stable, water-soluble, yet nevertheless lyophobic Au^0^ sols.

### 2.2. Structural Analysis of Au-Ar-Gal Nanocomposites

**Figure 2:**
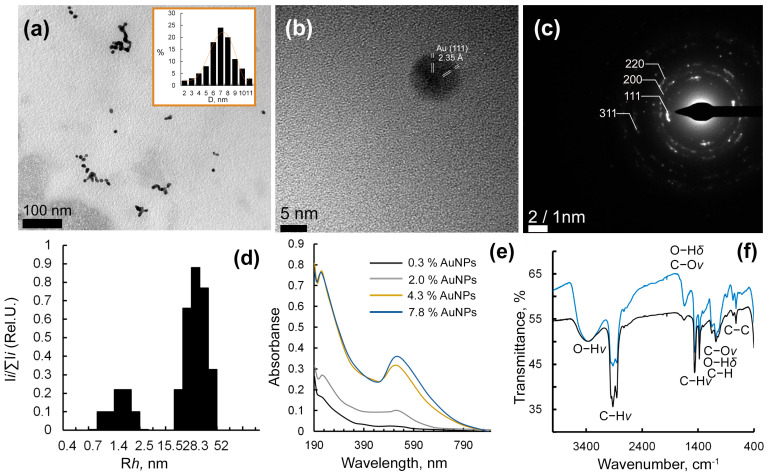
HR-TEM micrographs and dispersion distribution diagrams (inset) of Au nanoparticles in composites with gold contents 4.0%—(**a**); HR-TEM image Au nanoparticles with the fast Fourier-transform diffraction pattern of the nanoparticles in composites with gold contents 4.0%− (**b**); SAED image of single nanoparticles in composites with gold contents 4.0%—(**c**); Rh-distribution of 0.05% water solution of nanocomposite Ar-Gal-AuNPs containing 7.8% Au—(**d**); absorption spectra of 0.05% water solutions of Au-Ar-Gal nanocomposites—(**e**) and FTIR-spectra of native Ar-Gal (black) and Ar-Gal-AuNPs (7.8% Au) nanocomposite (blue)—(**f**).

According to the high-resolution transmission electron microscopy (HR-TEM) data, Ar-Gal-AuNPs nanocomposites are formed as particles that are distributed in the Ar-Gal polysaccharide matrix. These particles have a pronounced tendency to merge into chains due to their oriented coalescence (Figure 2a). The average size of the nanoparticles is primarily determined by the quantitative content of gold in the composite, varying in the range of 5.3–14.0 nm. The spherical shape of the nanoparticles is probably due to the spontaneous reduction of surface energy of the forming particles and the absence of conditions for their anisotropic growth in the reaction medium. The internal microstructure of the nanoparticles was investigated further using HR-TEM. The images obtained confirm the presence of mutually oriented lines, which obviously indicate the crystalline nature of the visualized AuNPs (Figure 2b). The interplanar distances between adjacent lines of the crystal lattice are 0.235 nm, corresponding to (111) spatial orientation of zero-valent gold crystallites (PDF #04-0784). Additionally, the electron diffraction pattern in the selected region of Ar-Gal-stabilized AuNPs demonstrates a set of point reflections, indicating the polycrystalline nature of the nanoparticles (Figure 2c). Analysis of the location of the point reflections and the measurement of the interplanar distances also confirms their belonging to the reflections of zero-valent gold.

According to the DLS data, the scattering intensity distribution of the aqueous solutions of Au nanoparticles stabilized by Ar-Gal is characterized by a bimodal fraction distribution (Figure 2d). Thus, two particle fractions with hydrodynamic radii (Rh) of 1.7 nm and 28.3 nm were detected in the nanocomposite aqueous solution sample containing 7.8% Au (Figure 2d). The first fast fraction of particles, with a Rh value of 0.9–2.0 nm that is close to Rh value the initial Ar-Gal fast fraction (2.7 nm) [35], presumably corresponds to individual Ar-Gal macromolecules in the form of a ball in an aqueous medium. The second fraction of particles, with an average Rh of 28.3 nm, most likely belongs to intermolecular associations of club-shaped Ar-Gal macromolecules with AuNPs formed within them. Variation in the quantitative content of AuNPs in the nanocomposite from 0.3% up to 2.0%, 4.3%, and 7.8% is accompanied by the preservation of the bimodal character of nanoparticle distribution with variation in the average Rh values of the fast and slow fractions at 2.5–4.7 nm and 28.7–34.7 nm, respectively. The appearance of an intense maximum of plasmonic absorption at 515–540 nm in the spectra of aqueous solutions of Au-Ar-Gal nanocomposites confirms the spherical geometry of the nanoparticles and the zero-valent state of gold in their composition (Figure 2e). The composite nature of the formed nanomaterials is also confirmed by FTIR-spectroscopy data. According to the spectra shown in Figure 2f, all bands characteristic of the initial Ar-Gal are preserved in the obtained nanocomposites. In particular, the FTIR-spectra of the initial Ar-Gal and its nanocomposite show a broad band in the region of 3374–3360 cm^−1^ corresponding to the valence vibrations of the O-H groups; intense bands at 2854–2924 cm^−1^ attributable to the valence vibrations of the C-H groups; a band at 1644 cm^−1^ belonging to the deformation vibrations of the O-H groups and the valence vibrations of the C-O groups. High-intensity narrow bands observed at 1455–1080 cm^−1^ correspond to the valence vibrations of the C-H and C-O groups, as well as the deformation vibrations of the O-H groups. The bands at 720–774 cm^−1^ are assigned to the deformation vibrations of the C-C bonds in the carbohydrate cycle. The increased intensity of the bands at 3360–3374 cm^−1^ and 1644 cm^−1^ may be attributable to hydroxyl groups being involved in formation of intermolecular associations via hydrogen bonds. Additionally, this increase may be associated with the growth of the carbonyl groups formed through the oxidation of the arabinogalactan by gold ions.

### 2.3. Assessment of Radical-Binding Capacity of Ar-GalAuNPs Composites by Chemiluminescent Method

**Figure 3:**
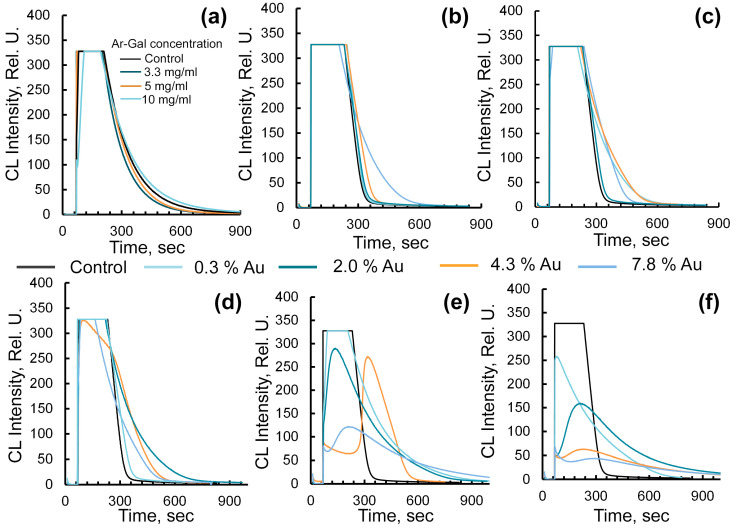
Luminol-activated chemiluminescence curves of the radical-generating system “horseradish peroxidase-H_2_O_2_” in the presence of native Ar-Gal—(**a**); Ar-Gal-AuNPs composites with varying amounts of gold in the composition and concentrations of aqueous solutions: 1 mg/mL—(**b**), 2.1 mg/mL—(**c**), 3.3 mg/mL—(**d**), 5 mg/mL—(**e**), 10 mg/mL—(**f**). *p* < 0.05, *n* = 3.

The antioxidant properties of the obtained nanocomposites were characterized using luminol as a chemiluminescence (CL) activator and the radical-generating system “horseradish peroxidase—H_2_O_2_”, as well as processing the CL kinetic curves using the TAR (Total Antioxidant Reactivity) method [39]. The intensity of the antioxidant effect of the tested samples was evaluated using the CL quenching degree (q), which was calculated as the ratio of the difference in CL intensity between the control sample and the sample with the tested potential antioxidant, divided by the CL intensity of the control sample. It was found that the addition of the obtained Ar-Gal-stabilized AuNPs to the radical-generating system “horseradish peroxidase—H_2_O_2_” was accompanied by a significant (up to 80 ± 6% compared to the control, *p* < 0.05) quenching of CL (Figure 3a–e).

At the same time, the fixed changes in CL kinetics were determined by the percentage of gold in the composites and their aqueous solution concentration. It is known that CL oxidation of luminol to the excited aminophthalate molecule, accompanied by photon emission in the presence of the radical-generating system ‘horseradish peroxidase–hydrogen peroxide’, is triggered by the interaction of horseradish peroxidase with hydrogen peroxide, producing the oxidized product with Fe^4+^ = O center and porphyrin cation radical [40]. The obtained product is then reduced to the initial peroxidase as a result of interaction with the luminol molecule, forming luminol radical and aminophthalate. Thus, the start of free radical generation process in the control sample is identified by a sharp increase in the intensity of CL, reaching a stationary luminescence phase within 3.5 min. The presence of this plateau indicates a constant rate of formation of radicals that initiate luminol oxidation. These radicals are formed during the decomposition of hydrogen peroxide by horseradish peroxidase. After the specified time has elapsed, the system enters a phase of rather sharp decline in CL intensity (within 2 min) due to a decrease in the total number of radicals in the system. Figure 3a shows the curves of CL kinetics for a radical-generating system to which pure Ar-Gal has been added. It was found that the introduction of Ar-Gal into an aqueous solution at a concentration of 3.3–5 mg/mL does not significantly alter the dynamics of CL intensity. When aqueous solutions of Ar-Gal-stabilized AuNPs were added to the analyzed radical-generating system ‘horseradish peroxidase—H_2_O_2_’, CL intensity changes are similar. In particular, the addition of an aliquot of 1–3.3 mg/mL of an aqueous solution of Ar-Gal-AuNPs is characterized by the preservation of the kinetic curve type, i.e., a sharp rise in CL intensity during the initial period of the experiment, followed by a transition to a plateau within 3.5 min, and a subsequent gradual decline to background values within 11 min (see Figure 3b–d). The CL intensity did not decrease at this time compared to the control. However, when the gold content of the composite was high (7.8%), a 2.6-fold decrease (±0.3, *p* < 0.05) in the CL intensity plateau duration was observed. The most pronounced change in CL kinetics was recorded when using aqueous solutions with a composite concentration of 5 and 10 mg/mL (Figure 3e,f). Using aqueous solutions of composites containing 0.3% and 2% Au at a concentration of 5 mg/mL, changes in CL kinetics included either a 1.4-fold decrease (±0.27, *p* < 0.05) in the duration of the CL intensity plateau or a 20% reduction in CL intensity without a plateau (compared to the control). An increase in the quantitative content of gold in the composite up to 4.3% and 7.8% was accompanied by significant CL quenching (up to 80% ± 6% compared to the control, *p* < 0.05) in the initial time period (up to 4 min). After this period, the CL intensity increased to 37–81% of the control level, declining gradually and smoothly to baseline values. The most significant changes in CL kinetics, and consequently the suppression of free radical generation in the ‘horseradish peroxidase—H_2_O_2_’ system, were observed when using aqueous solutions of nanocomposites at a concentration of 10 mg/mL. Even in conditions of the lowest gold content in the composite (0.3%), a 77% decrease in CL intensity (along with the disappearance of the plateau) compared to the control was recorded (Figure 3f). Conversely, an increase in the quantitative content of gold in the composite up to 2.0–7.8% was accompanied by significant (up to 80%) CL quenching in the initial period of up to 4 min, followed by a gradual, slight increase in CL intensity to 13–62% of the control value, with a gradual, smooth decline to baseline values (Figure 3f). Analysis of the experimental data on the radical-binding effect of the potential antioxidant Ar-Gal-Au composites indicates their lower intensity compared to the antiradical activity of silver nanoparticles. Practically complete quenching of CL (up to 87 ± 6.5%, *p* < 0.05) was reached only when testing solutions with a concentration of 10 mg/mL and a maximum amount of gold in the composite of 7.8%. This can probably also be attributed to the latent period in the action of the nanocomposites, which characterizes them as antioxidants of medium strength. Returning to the values of the degree of quenching (q), it can be concluded that these depend on the concentration of the aqueous nanocomposite solution, varying within the range 0.22–0.87 (±0.017–0.065), as well as on the quantitative content of gold nanoparticles in the composite. It was found that the minimum concentration that inhibits 50% of radical formation (IC_50_) in the system decreases with an increasing concentration of aqueous nanocomposite solutions, as well as with an increased amount of gold in their composition. Given the absence of antiradical action in pure Ar-Gal (Figure 3a), whose aqueous solutions are only able to neutralize 7.8 ± 0.72% (*p* < 0.05) of free radicals in the system even at a high concentration of 10 mg/mL, it may be deduced that the antiradical activity of gold nanocomposites is solely due to the AuNPs. Thus, the IC_50_ values of the nanocomposites (15.0–4.0 mg/mL) indicate that 50% inhibition of the CL radical-generating system requires the participation of 9·10^−9^–6·10^−8^ mol of gold.

The antioxidant properties of the Ar-Gal-stabilized AuNPs with different percentages of the inorganic phase to Ar-Gal were also evaluated using the CL analysis method using 2,2′-azobis (2-amidinopropane) dihydrochloride (ABAP) as a source of hydrophilic radicals to obtain high and long-lasting chemiluminescence. Thus, peroxyl radicals formed as a result of ABAP thermolysis interact with luminol (LH2) to form luminol radicals (LH•), from which aminophthalic acid molecules in an electron-excited state are formed through the formation of intermediates (luminol hydroperoxide and luminol endoperoxide).

ROO• + LH2 → ROOH + LH•

The antioxidant properties of the Ar-Gal-stabilized AuNPs with different percentages of the inorganic phase to Ar-Gal were also evaluated using the CL analysis method with 2,2′-azobis (2-amidinopropane) dihydrochloride (ABAP) as a source of hydrophilic radicals to obtain high and long-lasting chemiluminescence. Thus, peroxyl radicals, formed via thermolysis of ABAP with luminol (LH2), give luminol radicals (LH•). Aminophthalic acid molecules in an electron-excited state are then produced through the intermediate luminol hydroperoxide and luminol endoperoxide.

ROO• + LH2 → ROOH + LH•

**Figure 4:**
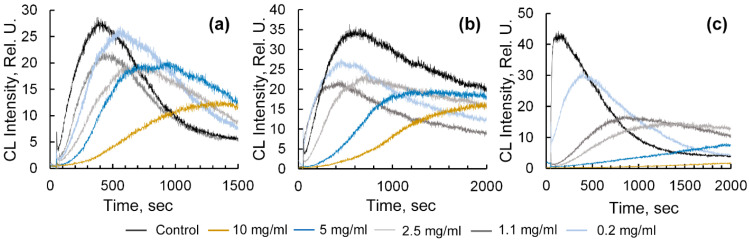
Effect of aqueous solutions (concentrations are shown in the figure) of gold nanocomposites containing 0.3% Au—(**a**), 2.0% Au—(**b**), and 7.8% Au—(**c**) on the chemiluminescence of the ABAP-luminol system. *p* < 0.05, *n* = 3.

To assess the antioxidant effect of the tested samples, the degree of q and the antioxidant capacity, calculated from the change in area (∆S) under the chemiluminescence kinetic curve (light sum) with the addition of a potential antioxidant, were used. The ∆S was calculated as the ratio of the difference between the area under the CL curve for a system without an antioxidant (S0) and the area under the chemiluminescence kinetic curve for a system with a pre-introduced antioxidant (Sa), divided by S0. The introduction of the obtained Ar-Gal-stabilized AuNPs into the radical-generating system “ABAP-luminol” was found to be accompanied by significant CL quenching (Figure 4a–c).

Introducing aliquots of nanocomposite aqueous solution containing 0.3% AuNPs into the analyzed sample decreases the CL intensity at its maximum value and increases the q parameter, ranging from 0.04 to 0.55, depending on the concentration of the aqueous Ar-Gal-AuNPs solution. The least pronounced radical-suppressing effect (q = 0.04) was observed when using a composite solution with a low concentration (0.2 mg/mL). An increase in the concentration of the composite aqueous solution to 1.1–10 mg/mL more significantly decreases the CL intensity at its maximum and raises the q parameter to 0.2–0.55. At the same time, the decrease in the area under the CL curve reaches only 63 ± 7.2% (*p* < 0.05) of the control. Maximum CL quenching was observed when introducing aqueous solutions of composites containing 7.8% gold into the analyzed sample volume. In this case, the decrease in CL intensity and the q parameter value reached 0.3–0.96 for the composite aqueous solution at concentrations ranging from 0.2 mg/mL to 10 mg/mL. Meanwhile, the decrease in the value of the total light sum recorded by the device from the analyzed sample, as characterized by the area under the CL curve, was 95.4 ± 8.4% (*p* < 0.05) for the most concentrated solution (10 mg/mL) (Figure 4c). These data also confirm the above results obtained with the radical-generating horseradish peroxide-hydrogen peroxide-luminol system, and demonstrate the pronounced radical-binding activity of Ar-Gal-stabilized AuNPs. This activity depends on both the percentage of nanoparticles in the nanocomposite and the concentration of the nanocomposite aqueous solution. According to modern concepts, the antiradical action of AuNPs is most likely based on their ability to act as electron donors for free radicals, reducing them to less active non-radical compounds [18]. Consequently, the larger quantity of AuNPs in the composite and in an aqueous solution, as well as their smaller size (a larger specific surface area) will contribute to electron abstraction from the nanoparticle surface and single-electron radical regeneration.

### 2.4. Creation and Characterization of Structural, Mechanical and Rheological Properties of Hydrogels Based on Ar-Gal-Stabilized AuNPs and κCG

#### 2.4.1. Assessment of Water-Holding Capacity of κCG-Ar-Gal-AuNPs Hydrogels

**Figure 5:**
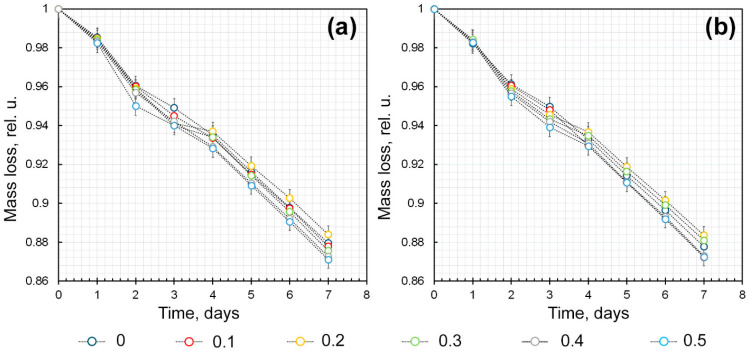
Water-holding capacity of hydrogels based on κCG-Ar-Gal-AuNPs system in the absence—(**a**) and presence—(**b**) of Ca^2+^ ions. Error bars are hidden in the bar when not visible; data are mean ± SD (0.015–0.021), *p* < 0.05, *n* =3.

The biomedical use of hydrogels containing AuNPs, including as wound-healing and anti-inflammatory agents with local action, is based on their ability to maintain the moisture content of the treated surface at an optimal level. This prevents the surface from drying out while allowing water-soluble AuNPs to be released from the hydrogel matrix. This would not be possible under conditions of hydrogel-to-xerogel transitions. Therefore, hydrogels intended for medical applications must have a high water-holding capacity. The water-holding properties of hydrogels can be ensured by selecting the ratio of the components included in their composition, combining polysaccharides, or introducing crosslinking agents. It was found that, 7 days after the start of the experiment, the relative moisture loss of κCG-Ar-Gal-AuNPs hydrogels varied from 11.6% to 12.9% of their initial mass (Figure 5a). The introduction of calcium ions at a concentration of 0.03%vol into the mixture does not increase in the water-holding capacity of the hydrogels (Figure 5b). The average water mass loss was 12.32% in this case versus 12.33% in hydrogels produced without the addition of calcium ions. The values obtained are almost identical, and the slight difference detected falls within the margin of error.

A change in the Ar-Gal-AuNPs/κCG ratio has a more significant effect on the water-holding capacity of hydrogels (*p* < 0.05, *n* =3). Thus, hydrogels with a ratio of Ar-Gal-AuNPs/κCG of 0.2 or 0.3 demonstrated the most pronounced moisture-retaining ability. This effect was also observed when calcium ions were introduced into the hydrogel. In this case, the moisture loss was 11.60 ± 0.77% and 11.63 ±0.79%, respectively (*p* > 0.05, *n* = 3). Deviations from this ratio, whether decreasing to 0.1 or increasing to 0.3–0.5, are characterized by high mass losses, reaching 12.04–12.90 ± 0.84–0.88% and 11.90–12.77 ± 0.95–0.88% (*p* < 0.05, *n* =3) for κCG-Ar-Gal-AuNPs and κCG-Ar-Gal-AuNPs-Ca^2+^ hydrogels, respectively.

#### 2.4.2. Assessment of the Swelling of κCG-Ar-Gal-AuNPs Hydrogels

**Figure 6:**
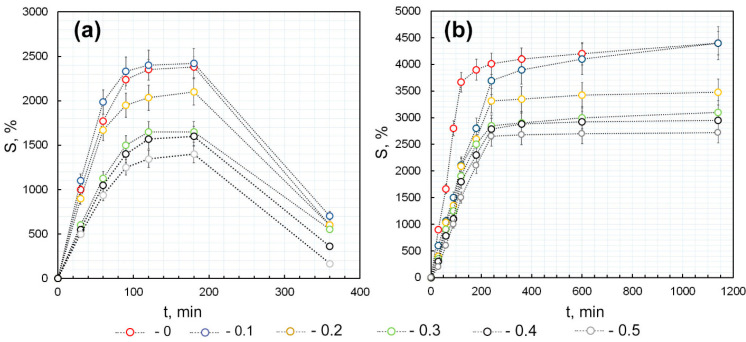
Kinetic curves of sorption of water by κCG-Ar-Gal-AuNPs—(**a**) and κCG-Ar-Gal-AuNPs-Ca^2+^—(**b**) gels depending on the Ar-Gal-AuNPs/κCG ratio; (*n* = 3, *p* < 0.05).

The effect of the Ar-Gal-AuNPs/κCG ratio, as well as the addition of a crosslinking agent (calcium ions), on the swelling ability of dried hydrogels (xerogels) was evaluated. As shown in Figure 6a, κCG-Ar-Gal-AuNPs hydrogels exhibit unlimited swelling owing to the absence of chemical or ionic crosslinking of the κCG gel-forming macromolecules. A transition to non-equilibrium swelling was observed for all hydrogels, regardless of the Ar-Gal-AuNPs/κCG ratio (*n* = 3, *p* < 0.05) (Figure 6a). A small increase in the swelling value (3 ± 0.16%, *p* < 0.01, *n* = 3) compared to the κCG-based hydrogel was revealed only when a small amount of the Ar-Gal-AuNPs composite was introduced to the κCG polymer matrix at a volume ratio of 0.1. Increasing this ratio from 0.1 to 0.5 reduced the swelling value (S, %) from 2420 ± 145% to 1440 ± 93.6% (*p* < 0.05, *n* = 3). In general, the dynamics of the increase in the swelling value for all the obtained hydrogels are uniform. High S values indicate the significant ability of hydrogels to adsorb water, primarily due to the hydration of hydrophilic groups of polysaccharides (bound water), followed by the diffusion of water molecules into the swollen polymer network to fill the space between the chains of the network or the pores and voids (free or bulk water).

The linear polysaccharide κCG can swell itself, whereas Ar-Gal, which has a branched, ridge-like structure, forms tangles through intra- and intermolecular hydrogen bonds in aqueous solutions and cannot swell. The κCG gel matrix lacks covalent or ionic crosslinking, which allows water molecules to penetrate the polymer network freely due to the osmotic pressure of its chains, resulting in infinite dilution. Presumably, when sufficiently hydrated, Ar-Gal diffuses into the solution in the pores of the already swollen κCG, destroying the gel’s spatial structure in the process. Decreased swelling is proportional to the volume fraction of the Ar-Gal-AuNPs composite relative to the κCG itself. This confirms the previously suggested assumption that the swelling ability is due solely to the κCG macromolecules. A slight increase in the S parameter with a small volume fraction of the Ar-Gal-based composite can probably be explained by the larger diameter of the pores and voids in the κCG macromolecular network, which was initially filled with Ar-Gal macromolecules during joint gelation and was subsequently emptied during hydrogel swelling. In theory, larger sizes of these voids would promote the penetration of additional of water molecules and increase the S parameter. However, higher amounts of the composite relative to κCG in the hydrogel would negate this effect (i.e., the growth of pores and voids). In contrast, this allows the predominance of the effect of a decrease in the volume fraction of κCG, as it is the only component capable of gelation and swelling. Nevertheless, such unlimited swelling of hydrogels significantly restricts their potential applications in biomedicine, including their use as local superabsorbent agents with additional pronounced biological activities due to their constituent components. The conversion of non-equilibrium swelling to equilibrium is possible using a cross-linking agent, particularly calcium ions. In this case, the calcium ions interact with the κCG macromolecules by forming cross-links between the negatively charged sulfogroups of the spiral-shaped macromolecules. This stabilizes the spatial structure of κCG and increases its rigidity, and the swelling of the hydrogel based on this composition becomes equilibrium-driven, which is preferable for biomedical use. Figure 6b shows experimentally obtained swelling curves of κCG-based hydrogels containing various ratios of Ar-Gal-AuNPs composite additives and a uniform concentration of calcium ions. The introduction of calcium ions into the κCG-Ar-Gal-AuNPs hydrogels composition was found to be accompanied by a significant increase of the swelling value (*p* < 0.01, *n* = 3), which was almost twice that of hydrogels without a crosslinking agent. Thus, the S value of all hydrogels varies 4400–2700 ± 308–188% of the mass of dried samples when calcium ions are introduced. In addition, the transition of these hydrogels from unlimited to limited swelling has been experimentally confirmed. Complete dissolution of the hydrogels was not observed even after 20 h after the start of the experiment (*p* < 0.05, *n* = 3). The swelling was most intense in the first 4 h after the start of the experiment (by 3700–2650 ± 296–172%, (*p* < 0.05, *n* = 3), followed by a slight increase in the S value over the next 16 h (700–50 ± 40–3.5%, *p* < 0.05, *n* = 3). The introduction of a composite based on Ar-Gal macromolecules into the composition of κCG changes the dynamics of the S value increase over time. The nature of this change and the S value of the κCG-AG-AuNPs-Ca^2+^ hydrogels were determined by the Ar-Gal-AuNPs/κCG ratio. Thus, the introduction of the composite into the Ca^2+^-crosslinked κCG matrix decreased the rate constant of water absorption by dried hydrogel samples during the first 4 h. The rate constant describing the water sorption by hydrogels was calculated using the data obtained from the angle of inclination of the linear section of the experimental curves. This constant was found to decrease from 30.76 for pure Ca^2+^-crosslinked κCG to 15.2, 14.4, 12.7, 11.5 L mol/s for composite AuNPs-containing hydrogels. This decrease in the water sorption rate of the hydrogels is presumably due to a slowdown in the diffusion of water molecules into the hydrogel’s structural cavities and the hydrogen bonding of Ar-Gal macromolecules with κCG macromolecules. Owing to its inability to swell, Ar-Gal interacts with water, leading to primary solvation, relaxation of chains, and an increase in their mobility. This is followed by dissolution and removal from the pores of the κCG hydrogel matrix and further penetration of water molecules into the vacated space inside the κCG matrix. Thus, the S value increases slowly due to the need for hydration, relaxation and dissolution of Ar-Gal, and its subsequent transfer into solution from the hydrogel matrix. After this, the κCG water sorption process continues. This assumption correlates well with the experimental data, which show that the decrease in the rate constant of the water sorption by κCG-Ar-Gal-AuNPs-Ca^2+^ hydrogels is inversely proportional to the Ar-Gal-AuNPs/κCG ratio. As in the case of Ca^2+^-unlinked hydrogels, lower maximum values of S for crosslinked gels is explained by a reduction in the amount of polysaccharide capable of swelling (κCG), which is caused by an increase in the Ar-Gal-AuNPs/κCG volume ratio.

#### 2.4.3. Evaluation of the Dynamics of AuNPs Release from the κCG-Ar-Gal-AuNPs Hydrogel

The biological activity of AuNPs, particularly their antioxidant properties, is primarily determined by their ability to interact with targets. This condition can only be satisfied by releasing gold nanoparticles from the polymer matrix of the hydrogel. Prolonged release is preferable as it preserves active concentrations of nanoparticles in the affected area. To determine the completeness and dynamics of the release of Ar-Gal-stabilized AuNPs from the κCG-Ar-Gal-AuNP hydrogel matrix, a spectrophotometric study of the diffusion of AuNPs into an aqueous solution was carried out. The dynamics of the nanoparticle release were assessed by measuring the change in the intensity of the maximum plasmon absorption in the region of 528 nm, which is characteristic of AuNPs. The absorption spectrum of an aqueous solution of Ar-Gal-AuNPs was preliminarily recorded, revealing a linear dependence of the absorption intensity at 528 nm on the concentration of the composite.

**Figure 7:**
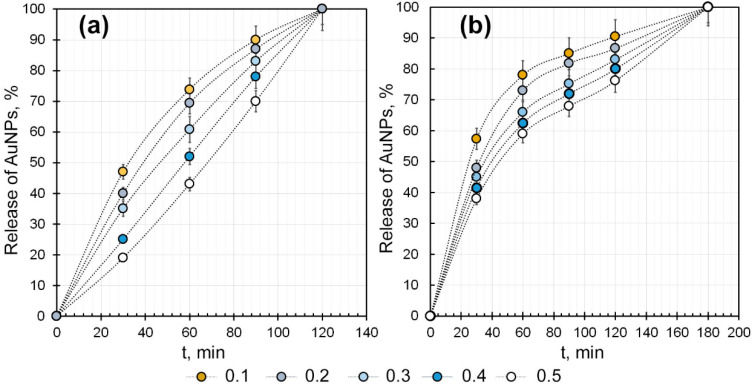
Dynamics of gold nanoparticle release from the matrix of the κCG-Ar-Gal-AuNPs—(**a**) hydrogel and the κCG-Ar-Gal-AuNPs—(**b**) Ca^2+^–crosslinked hydrogel, depending on the κCG/Ar-Gal-AuNPs ratio. Data are mean ± SD (1–7%), *p* < 0.05, *n* =5.

The observed significant changes in the dynamics of AuNP release depend on the type of hydrogel (unlinked or crosslinked). Due to the absence of rigid chemical or ionic crosslinking in κCG-Ar-Gal-AuNPs hydrogels, as well as their non-equilibrium swelling behavior, AuNP release was rather intensive (43–74 ± 3–5%; *p* < 0.05, *n* = 5) during the first 60 min after the hydrogel was placed in solvent. Subsequent exposure for 120 min resulted in further AuNP release, albeit at a reduced rate (57–26 ± 3–5, 4–2%; *p* < 0.05, *n* = 5), with complete dissolution of the hydrogel itself and 100% AuNP release occurring after 120 min (Figure 7a). Increasing the Ar-Gal-AuNPs/κCG ratio from 0.1 to 0.5 slowed the release rate of nanoparticles into the solvent, probably due to the need for relaxation processes during the solvation of Ar-Gal macromolecules within the κCG structural network, and the destruction of hydrogen bonds between these macromolecules, which increases their mobility before the direct release of Ar-Gal-stabilized nanoparticles into the solvent. Introducing calcium ions into the κCG-Ar-Gal-AuNPs hydrogel composition changed the dynamics of AuNP release. The highest percentage of released nanoparticles (59–78 ± 4–5.6%; *p* < 0.05, *n* = 5) was observed one hour after the hydrogel was placed in water (Figure 7b). The release of nanoparticles then slowed down significantly, reaching 100% only by the end of the sixth hour. The presence of ionic crosslinking between the κCG molecules probably significantly complicated the diffusion of the composite from its pores. This resulted in the prolonged release of AuNPs and maintenance of their effective concentrations at the required level over a longer period of time. An increase in the Ar-Gal-AuNPs/κCG ratio also slowed release of AuNPs from the hydrogel matrix, likely owing to more Ar-Gal macromolecules participating in forming hydrogen bonds with κCG macromolecules, which must be cleaved before Ar-Gal-AuNPs can be released into an aqueous solution. In addition, the reduction in the diffusion rate of AuNPs from the hydrogel matrix pores into an aqueous solution may be attributable to a change in their concentration gradient. Thus, in the initial period (up to 60 min) after placing the hydrogel in water, the gradient was maximal since it assumes the presence of very high concentrations of the composite inside the hydrogel matrix and the complete absence of Ar-Gal-AuNPs in the water. After 43–78% of the Ar-Gal-AuNPs entered the aqueous solution, however, these concentrations equalized to some extent and the intensity of their diffusion into the solution decreased.

#### 2.4.4. Determination of the Elasticity Modulus of κCG-Ar-Gal-AuNPs Hydrogels Crosslinked by Calcium Ions

**Figure 8:**
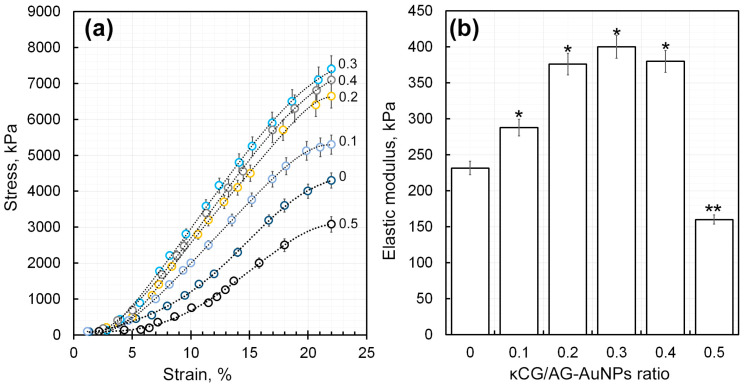
Effect of AG-AuNPs/κ-CG ratio (the ratio is shown in the figure) on the stress–strain dependence of hydrogels—(**a**) and their elastic modulus—(**b**). The Ar-Gal-AuNPs/κCG ratio is shown in the figure. * *p* < 0.05, ** *p* < 0.01, *n* = 3.

Based on the above data, Ca^2+^-crosslinked hydrogel compositions based on κCG-Ar-Gal-AuNPs were found to have great potential for practical application due to their more pronounced swelling ability and its limited type, as well as the prolonged release of AuNPs from their matrix. Consequently, a uniaxial compression investigation into the effect of the Ar-Gal-AuNPs-κCG ratio on the value of the Young’s modulus and the stress–strain dependence for Ar-Gal-AuNPs-κCG hydrogels was conducted exclusively for Ca^2+^-crosslinked hydrogels. The results are shown in Figure 8. The introduction of an aqueous solution of the Ar-Gal-AuNPs nanocomposite into the κCG polysaccharide matrix in the presence of calcium ions was accompanied by an increase (*p* < 0.05, *n* =3) in the Young’s modulus of the resulting hydrogel composition, probably owing to a higher number of crosslinks in the hydrogel structure. This increase can be explained by both Ca^2+^-induced ion crosslinking of κCG macromolecules, and the non-covalent interaction of Ar-Gal macromolecules stabilizing the surface of AuNPs with κCG macromolecules. This increase in the Young’s modulus was observed for hydrogels with an Ar-Gal-AuNPs/κCG volume ratio ranging from 0.1 to 0.3. The Young’s modulus increases by a factor of 1.2–1.7 ± 0.06–0.1 (*p* < 0.05, *n* =3) compared to pure κCG. However, with an increase in this ratio to 0.4 and 0.5, the Young’s modulus value drops significantly. At the highest ratio of 0.5, it is only 65 ± 2% (*p* < 0.01, *n* = 3) of the initial pure κCG Young’s modulus value. The reason for this decrease in elastic modulus in hydrogels containing a large amount of the Ar-Gal-AuNPs nanocomposite is probably the formation of a large volume of pores and voids in the internal spatial structure of the gel.

Thus, the non-covalent interaction of a large number of Ar-Gal macromolecules with κCG (including through hydrogen bonds) decreases the amount of Ca^2+^-induced ion crosslinking between κCG sulfogroups. This is due to stronger spatial hindrances and to the fact that some of the carrageenan sulfate groups are screened by Ar-Gal macromolecules that are not involved in the formation of these ion crosslinks. Consequently, gels exhibit reduced strength when the Ar-Gal-AuNPs/κCG ratio is high. Nevertheless, the highest values of Young’s modulus and deformation were observed in hydrogels with component ratios in the range 0.2–0.4, where sufficient stress was applied. In other words, the introduction of calcium ions and a composite based on the arabinogalactan polysaccharide matrix into the κCG matrix can significantly modify the most important rheological properties of the resulting hydrogels, particularly their elasticity and strength.

## 3. Conclusions

In conclusion, aggregatively stable nanocomposites consisting of AuNPs with an average size of 7.6–11.6 nm and Ar-Gal were obtained by exploiting the reducing and stabilizing properties of the natural polysaccharide Ar-Gal. The synthesized composites were characterized in detail using a variety of modern spectral and microscopic research methods. The valence state of gold in the nanocomposites, their size distribution and the size of the nanoparticles in an aqueous solution were determined. The radical-binding properties of the nanocomposites were examined for the first time using the chemiluminescent method and two radical-generating systems: ‘horseradish peroxidase-hydrogen peroxide-luminol’ and ‘ABAP-luminol’. It was found that these properties are primarily determined by the AuNPs themselves and their amount in the composite. Composite hydrogels with different volume ratios of components were produced for the first time from the available natural polysaccharide κCG and the original Ar-Gal-stabilized AuNPs. The ratio of these components was shown to define the hydrogels’ water-holding capacity, swelling ability, ability to release gold nanoparticles into solution, and modulus of elasticity. It was confirmed experimentally that hydrogels with a volume ratio of Ar-Gal-AuNPs/κCG of 0.1–0.3 exhibited the most pronounced swelling ability, prolonged gold nanoparticle release, and the highest modulus of elasticity. A decrease in these parameters is due to an excess of this ratio. The valuable antiradical properties of the synthesized Ar-Gal-AuNPs nanocomposites, coupled with the promising rheological properties of the resulting hydrogels, suggest their potential application as complex anti-inflammatory drugs with a local action, including those with a pronounced wound-healing effect.

## 4. Materials and Methods

### 4.1. Materials

The arabinogalactan of Siberian larch (*Larix sibirica*) isolated from natural raw materials by aqueous extraction and purified according to the protocol described in [38] was used. Found, %: C-41.9, H-7.4, O-50.7. κ-Carrageenan WR-78 mark (Mw 1800 kDa, CP Kelco, Lille Skensved, Denmark) which was previously subjected to partial alkaline depolymerization according to the procedure described in detail in [37]. The resulting κ-CG sample exhibited improved water solubility and reduced Mw value to 1100 kDa. κ-CG: Found, %: C 32.04; H 6.10; O 52.44; S 6.08; K 3.34; Na 3.67. IR spectra (KBr, ν, cm^−1^): 3560, 3422 (OH), 2970, 2942, 2913 (C-H), 1200–1000 (C-O), 910, 771 (β-glycosidic bond of pyranose rings), 850 (SO_3_). All commercial reagents HAuCl_4_, NaOH, KOH, KH_2_PO_4_, ethanol—Reachim, horseradish peroxidase (Sigma Aldrich, St. Louis, MA, USA), 30% hydrogen peroxide (Sigma Aldrich, St. Louis, MA, USA), luminol (Sigma Aldrich, St. Louis, MA, USA), ABAP ((Sigma Aldrich, St. Louis, MA, USA) were used without further purification.

### 4.2. Methods

#### 4.2.1. Transmission Electron Microscopy

High-resolution transmission electron microscopy was performed on a Tecnai G2 20F S-TWIN FEI microscope (FEI Europe B.V., P.O., Eindhoven, The Netherlands) using formvar copper grids as a substrate for a layer of Ar-Gal-stabilized gold nanoparticles. A 0.05% aqueous solution of nanocomposites was used to prepare the sample. A drop of this solution was placed on the surface of the mesh and dried at room temperature under vacuum. The particle size measurement was carried out manually using the IPWin45 program by processing of the obtained HR-TEM micrographs. To obtain the size distribution, multiple images with a total particle count of at least 500 were used for each composite. Indexing of reflections visualized in the electron diffraction patterns of the resulting nanocomposites was performed using Gatan software (Version 3.01.598.0 Gatan Inc. Digital Micrograph, Pleasanton, CA, USA).

#### 4.2.2. Elemental Analysis

The elemental composition of nanocomposites was determined using a Hitachi TM 3000 electron scanning microscope with an SDD XFlash 430-4 X-ray detector by X-ray energy dispersive microanalysis (Angstrom Scientific Inc., Ramsey, NJ, USA) and a Thermo Scientific Flash 2000 CHNS analyzer (Thermo Fisher Scientific, Waltham, MA, USA).

#### 4.2.3. Dynamic Light Scattering

The hydrodynamic radii (Rh) of Ar-Gal and its Au-Ar-Gal nanocomposites were determined by dynamic light scattering (DLS) on a Photocor Compact-Z (Photocore, Moscow, Russia) correlation spectrometer (light source—thermostabilized semiconductor laser of 20 mW power with wavelength λ = 638 nm) at an angle of 90°. The correlation function was analyzed using Dynals dynamic light scattering data processing program. Solutions for analysis were prepared by dissolving at room temperature for at least ten hours 5 mg of sample in 5 mL of distilled water, pre-filtered through a syringe filter. The time for each measurement was at least 200 s. The measurement was performed in triplicate.

#### 4.2.4. Optical Spectroscopy

Optical absorption spectra of 0.05% aqueous solutions of nanocomposites were recorded on a Perkin Elmer Lambda 35 spectrophotometer (PerkinElmer, Waltham, MA, USA) against distilled water in a 1 cm quartz cuvette in the wavelength range of 190–1000 nm.

#### 4.2.5. FTIR-Spectroscopy

FTIR-spectra were recorded on a Bruker Vertex 70 FTIR spectrometer (RAM II) (Bruker, Ettlingen, Germany) using Vaseline oil in the 4000–400 cm^−1^ range.

### 4.3. The General Method of Nanocomposites Synthesis

Nanocomposites were synthesized according to the protocol described in detail in [31] with minor modifications. Briefly: 5 mL of an aqueous solution containing 0.004–0.145 g HAuCl_4_ was added to 50 mL of aqueous Ar-Gal solution (0.0175 g/mL) at 35 °C and intense stirring. The mixture was kept at 35 °C for 30 min, after which the pH of the medium was increased to 10.6 by adding an aqueous solution of 1N NaOH followed by increasing the temperature of the reaction medium to 70 °C on a water bath. The duration of synthesis varied depending on the amount of the introduced precursor from 20 to 50 min and was determined by stabilization of the absorption spectrum (no increase of the plasmonic absorption intensity) and stabilization of the reaction medium pH (no decrease of pH during synthesis, indicating the completion of the metal reduction process). The main variable synthesis parameters are shown in Table 1. The isolation of the target nanocomposite and its purification from impurities was carried out by precipitation of the reaction medium in a fourfold excess of EtOH and repeated washing of the formed violet-colored precipitate with ethanol followed by air drying at room temperature. The samples were dried to natural humidity (5–6%) and stored in borosilicate glass containers with UV protection in an atmospheric air at a temperature of 5–7 °C.

EA: Au-Ar-Gal (0.3% Au), Found (%): C, 40.2; H, 5.87, O-53.63; Au, 0.3. Au-Ar-Gal (2.0% Au), Found (%): C, 40.78; H, 5.52; Au, 2.0, O-51.70. Au-Ar-Gal (4.3% Au), Found (%): C, 39.62; H, 5.0; Au, 4.3, O-51.08. Au-Ar-Gal (7.8% Au), Found (%): C, 39.46; H, 4.95; Au, 7.8, O-47.79.

### 4.4. Assessment of Radical-Binding Ability of Gold Nanoparticles

#### 4.4.1. Evaluation of Antiradical Activity Using the Radical-Generating System «Horseradish Peroxidase-Hydrogen Peroxide-Luminol»

The radical-binding ability of Au-Ar-Gal composites was evaluated according to the modified methodology presented in detail in [27] using a Lum-100 chemiluminometer (DIsoft, Moscow, Russia) and PowerGraph 3.3 software for chemiluminescence registration. Briefly: 40 μL of 1 mM luminol dissolved in phosphate buffer (pH 7.4), 40 μL of aqueous solution of Au-Ar-Gal nanocomposites in concentrations (1–10 mg/mL) (or solutions of Ar-Gal or HAuCl_4_ and ascorbic acid for reference) and 920 μL of phosphate buffer were placed in the cuvette. After recording the background chemiluminescence signal for 40 s, 100 μL of H_2_O_2_ at a concentration of 1 mM was added to the cuvette with the analyzed mixture and continued to record the CL for 15 min. The experiment was performed in triplicate (*n* = 3) for each sample. The obtained data were averaged and presented in the graphs shown in Figure 3. All measurements were carried out with the following device parameters: recording frequency—1 Hz, sensitivity 100,000 Pps, temperature—23.5 °C, step—1 s, measurement time—15 min.

#### 4.4.2. Study of the Radical-Binding Ability of Composite Using the «ABAP-Luminol» Radical-Generating System

A plastic cuvette was filled with 900 μL of phosphate buffer (pH 7.4), 20 μL of 0.03 mM luminol solution in phosphate buffer (pH 7.4), 40 µL of an aqueous solution of Ar-Gal-AuNPs nanocomposites or control solutions (ascorbic acid and native Ar-Gal) in concentrations (0.2–10 mg/mL). Next, the cuvette was placed in a chemiluminometer, thermostatted for 10 min at 37 °C, and the intensity of the baseline chemiluminescence of the sample was recorded. After recording the background CL signal for 40 s, 40 µL of 0.16 mM ABAP solution in buffer was added to the cuvette with the analyzed mixture, and CL recording was continued for 25 min. The experiment was performed in triplicate (*n* = 3) for each sample. The obtained data were averaged and presented in the graphs shown in Figure 4. All measurements were carried out with the following device parameters: recording frequency—1 Hz, sensitivity 100,000 Pps, temperature—37 °C, step—1 s, measurement time—25 min.

The degree of chemiluminescence quenching q was calculated using equation:

$$q=\frac{I0-Ii}{I0}$$

where I0 is the value of the maximum chemiluminescence intensity in the control (without of antioxidant), Ii is the value of the maximum chemiluminescence intensity in the experimental sample with the addition of a potential antioxidant or standard (ascorbic acid).

Antioxidant capacity was also calculated from the measured light sum values in the control and experimental samples using the method described in detail in [27].

The minimum inhibitory concentrate (mg/mL) of the nanocomposite required for 50% scavenging of free radicals in the system (IC50) was calculated from equation:

$$IC50=\frac{S0-Si}{S0}\times 100\%$$

where S0 is the value of the light sum (V·s) emitted during 15–25 min of data recording after the introduction of hydrogen peroxide in the control (without antioxidant). Si is the value of the light sum (V·s) emitted during 15–25 min of data recording after the introduction of hydrogen peroxide in the experimental sample (with a potential antioxidant or ascorbic acid) or triggering the process of generating free radicals as a result of thermolysis.

### 4.5. Preparation of Ar-Gal-AuNPs-κ-CG Hydrogels

To create the hydrogel composition, an Ar-Gal-AuNPs nanocomposite sample containing 4.0% gold was used, namely, its 0.029 g/mL aqueous solution and an aqueous (0.029 g/mL) solution of the κCG polysaccharide. Hydrogels were obtained by mixing on magnetic stirrer at 35 °C for 3 h aqueous solutions of Ar-Gal-AuNPs composites and κCG in different Ar-Gal-AuNPs/κ-CG ratios varying from 0 (only κ-CG) to 0.2, 0.3, 0.4, 0.5 in order to obtain samples with satisfactory water-holding capacity, swelling capacity, and to modify the strength and elasticity of hydrogels. The main mixing parameters of the components for the production of composite hydrogels of Ar-Gal-AuNPs-k-CG are given in Table 2. After mixing, the tubes with mixture were placed in a thermostat (temperature 7 °C) for gel formation. In addition, series of hydrogels (No. 7–12 in Table 2) with similar ratio of components but addition of calcium chloride in the amount of 0.078 mmol for each hydrogel sample was also obtained. The aliquot of the aqueous solution of calcium chloride was injected into finished mixtures of Ar-Gal-AuNPs-κ-CG. The gel formation was evaluated after 4 h.

### 4.6. Study of Water-Holding Capacity of Hydrogels

To evaluate the water-holding capacity of the obtained composite hydrogels, three parallel experiments were carried out with all samples of Ar-Gal-AuNPs-κ-CG hydrogels (and pure κ-CG for comparison) presented in Table 2. In this case, daily weighing of the samples to determine the dynamics of mass loss was carried out three times for each sample in all parallel experiments. The water-holding capacity of hydrogels was estimated gravimetrically by fixing the hydrogel mass loss during its storage in the thermostat at atmospheric pressure and moisture and temperature 30 °C. Measurements were carried out daily for 7 days at the same time. The processing of the obtained results was carried out on the basis of the protocol previously presented in [41]. The hydrogel mass loss curves during storage shown in Figure 5 were obtained by averaging the mass values obtained for each hydrogel sample in three parallel experiments and three measurements within each experiment for each sample. The reliability of the obtained results is indicated in Figure 5.

### 4.7. Study of AuNPs Release Dynamics from Hydrogels in Aqueous Medium

The dynamics of AuNP release from hydrogels of different compositions was investigated spectrophotometrically by detecting the intensity of the plasmon absorption band in aqueous solution containing the sample under study according to protocol described in [37]. The experiment was performed in five parallel replicates for each hydrogel sample. The curves for the release dynamics of gold nanoparticles from the hydrogel matrix shown in Figure 7 are the result of averaging the experimental values obtained from five parallel replicates. The reliability of the obtained results is indicated in Figure 7.

### 4.8. Investigation of Gel Swelling

The swelling study of the obtained composite hydrogels was carried out in three parallel experiments for each hydrogel sample presented in Table 2. In this case, the weighing of the samples to determine the dynamics of water sorption by hydrogels was carried out three times for each sample in all parallel experiments. The hydrogels were dried to a constant weight. Then the bulk of the dried hydrogel (xerogel) was immersed in distilled water at a temperature of 30 degrees Celsius. The mass of the swollen hydrogel was measured every 30 min. The experimental swelling value S was calculated as a relative increase in the water content in the hydrogel according to the following equation:

$$S\;\%=\frac{mt-m0}{m0}\times 100$$

where S is the swelling value (%), mt is the mass of the hydrogel sample at time (t), and m0 is the mass of the hydrogel sample before the experiment.

The swelling curves presented in Figure 6 are the result of averaging the experimentally obtained values of the mass of the swollen hydrogel for each sample from three parallel experiments.

### 4.9. Determination of the Elastic Modulus of Composite Hydrogels κ-CG-AG-AuNPs-Ca^2+^

All rheological measurements were performed in triplicate for each sample according to the methodology in [37] using a HAAKE Viscotester iQ rheometer (Thermo Scientific, Waltham, MA, USA) in a cell with parallel plate geometry. Hydrogel samples, 1.3 cm in diameter and 1.3 cm in height, pre-thermostated at the measurement temperature (room temperature) for 4 h were used for the study. The study was conducted in three parallel experiments. The data characterizing the elastic modulus of the obtained hydrogels, presented in Figure 8, are the result of averaging the experimental data from three parallel experiments and three measurements within a single experiment for each sample.

### 4.10. Statistical Analyses

Data are presented as means ± standard deviations (*n* = 3 or 5). One-way ANOVA with Tukey’s honest significance test was applied to determine statistically significant differences in independent measurements. Results were considered significant at * *p* < 0.05 and ** *p* < 0.01; *p* > 0.05 was not considered significant.

## Figures and Tables

**Table 1:** Synthesis conditions and yield of gold-containing nanocomposites.

Relation (g/g) HAuCl_4_/Ar-Gal	Synthesis Time, min	Yield, %	Au, %	Au Conversion, %
0.0047	20	83	0.3	100
0.035	35	79	2.0	100
0.070	42	71	4.3	100
0.17	50	48.5	7.8	100

**Table 2:** Conditions for the preparation of hydrogels.

	V κCG, mL	V Ar-Gal-AuNPs, mL	V Water, mL	Relation Ar-Gal-AuNPs/κ-CG	CaCl_2_ mmol	pH
1	2	0	1	0	-	7.10
2	2	0.2	0.8	0.1	-	7.08
3	2	0.4	0.6	0.2	-	6.98
4	2	0.6	0.4	0.3	-	7.11
5	2	0.8	0.2	0.4	-	7.0+
6	2	1.0	0	0.5	-	6.89
7	2	0	1	0	0.078	7.03
8	2	0.2	0.8	0.1	0.078	7.00
9	2	0.4	0.6	0.2	0.078	7.1
10	2	0.6	0.4	0.3	0.078	6.97
11	2	0.8	0.2	0.4	0.078	7.00
12	2	1.0	0	0.5	0.078	6.99

## Data Availability

Data are contained within the article.
